# Role of Baicalin in Anti-Influenza Virus A as a Potent Inducer of IFN-Gamma

**DOI:** 10.1155/2015/263630

**Published:** 2015-12-10

**Authors:** Ming Chu, Lan Xu, Ming-bo Zhang, Zheng-yun Chu, Yue-dan Wang

**Affiliations:** ^1^Department of Immunology, School of Basic Medical Sciences, Peking University, Beijing 100191, China; ^2^Key Laboratory of Medical Immunology, Ministry of Health, Beijing 100191, China; ^3^Pharmacy Departments, Liaoning University of Traditional Chinese Medicine, Liaoning 116600, China

## Abstract

Baicalin (BA) is a flavonoid compound purified from* Scutellaria baicalensis* Georgi and has been shown to possess a potent inhibitory activity against viruses. However, the role of BA in anti-influenza virus has not been extensively studied, and the immunological mechanism of BA in antiviral activity remains unknown. Here, we observed that BA could protect mice from infection by influenza virus A/PR/8/34 (H_1_N_1_), associated with increasing IFN-*γ* production, but presented no effects in IFN-*γ* or IFN-*γ* receptor deficient mice. Further study indicated that BA could inhibit A/PR/8/34 replication through IFN-*γ* in human PBMC. Moreover, BA can directly induce IFN-*γ* production in human CD4^+^ and CD8^+^ T cells and NK cells, and activate JAK/STAT-1 signaling pathway. Collectively, BA exhibited anti-influenza virus A (H_1_N_1_) activity* in vitro* and* in vivo* as a potent inducer of IFN-*γ* in major IFN-*γ* producing cells.

## 1. Introduction

Influenza is one of the most common respiratory diseases in human and is potentially lethal in immunocompromised persons. It affects all types of people, from infants to the elderly, and is usually caused by influenza virus A and influenza virus B. Influenza virus is an enveloped virus of the Orthomyxoviridae family, which has a unique capacity for genetic variation that is based in two molecular features of the virus family [1]. Because of the high degree of virus variability, cellular responses directed in particular against more conserved internal proteins have been investigated, involving CD4^+^ T cells and CD8^+^ T cells that are able to recognize epitopes shared by several subtypes [2, 3]. In cellular responses, IFN-*γ* producing cells can favor viral elimination from nasal tissues, and more generally, IFN-*γ* plays a critical role in cell-mediated immunity, stimulating NK cells and macrophages activity.

In mammals, the IFN system is a central innate antiviral defense mechanism, first identified in 1957 by Isaacs and Lindenmann during their seminal study on virus interference [4]. It was also the first cytokine to be purified to homogeneity, cloned, sequenced completely, and produced in recombinant form and in extensive clinical application [5]. IFN response represents an early host defense event, one that occurs prior to the onset of the adaptive immune response, produced both in the early stages of infection by NK cells and at later stages by activated CD4^+^ and CD8^+^ T cells [6]. The innate immune response to viral infection depends on the integrity of this network of cytokines, which is tightly regulated [7].

In the traditional Chinese medicine, one therapeutic use of* Scutellaria* is to treat cold, an illness usually caused by influenza virus infection. In 1990, a flavone isolated from the medicinal plant* Scutellaria baicalensis* Georgi was shown to possess a potent antiviral activity against influenza virus. Thereafter,* S. baicalensis* and its main constituents including BA (7-glucuronic acid, 5,6-dihydroxyflavone) were found to possess a potent inhibitory activity against various viruses [8–13]. In 2007, we first reported that the compound extract of* Scutellaria* could inhibit influenza virus replication and effect on inducing IFN-*γ* secretion* in vivo*, in which the content of BA was merely 7% [12]. Thereafter, we obtained pure BA from* Scutellaria* and confirmed its antiviral activity on mouse-adapted A/FM/1/47 influenza virus A (H_1_N_1_) in mice [13]. Nowadays, BA was documented to exert its anti-influenza activity by modulating viral protein NS1-mediated cellular innate immune responses [11]. In this paper, we further studied the direct effects of BA on immune systems, including CD4^+^ and CD8^+^ T cells and NK cells. We believe that further study in the antiviral activity of BA will aid in our ability to design effective interventions and treatments for viral prevention and therapy.

## 2. Materials and Methods

### 2.1. Animals

4–6-week IFN-*γ*  −/− knockout (KO), IFN-*γ* receptor −/− KO, and +/+ wild-type (WT) mice (all on a C57BL/6 background) were obtained from the Jackson Laboratory (Bar Harbor, ME) and kept in a pathogen-free environment.

### 2.2. Virus

Mouse-adapted strain of human influenza virus A/PR/8/34 (H_1_N_1_) was obtained from the Chinese Center for Disease Control and Prevention and adapted to growth in MDCK cells. Stock virus was stored at −80°C until use.

### 2.3. Reagent

BA was isolated and prepared by procedures elsewhere [12, 13], provided by Liaoning University of Traditional Chinese Medicine. Its structure has been determined to be 7-D-glucuronic acid-5-6-dihydroxyflavone (mp 223°C and *λ*max⁡276).

### 2.4. Cells

MDCK cells were obtained from ATCC and expanded at 37°C in a humidified atmosphere of 5% CO_2_ and 95% air in MEM (Invitrogen) containing 10% newborn calf serum (GIBCO-BRL). Mouse CD4^+^ and CD8^+^ T cells and NK cells were isolated to >95% purity by automated magnetic negative selection using EasySep mouse CD4^+^ and CD8^+^ T cells and NK cells enrichment kits (StemCell Technologies Inc.) from spleen cells of experimental C57BL/6 mice. Human PBMC were obtained by centrifugation heparinized blood on Lymphoprep gradient and recovered from the gradient and washed first with saline and twice in RPMI 1640 (GIBCO-BRL) plus glutamine-streptomycin-penicillin (GSP) and 5% fetal bovine serum (FBS). Human CD4^+^ and CD8^+^ T cells and NK cells were isolated to >95% purity by automated magnetic negative selection using EasySep human CD4^+^ and CD8^+^ T cells and NK cells enrichment kits (StemCell Technologies Inc.) from PBMC of healthy volunteers.

### 2.5. Experimental Infection and Treatment of Mice

Mice were infected intranasally with 0.1 LD_50_ (5 × 10^3^ PFU/mL) of A/PR/8/34 in 100 *μ*L PBS [14]. Infected mice were treated with various concentrations of BA at 1.0 g/kg, 1.5 g/kg, or 2.0 g/kg every 24 h for 14 days. Infected mice were then treated with the compound solutions prepared in PBS (pH = 7.2) by intragastric administration. Ten infected mice were used for each treatment and control regimen. Mouse survival rate was determined. Weight and clinical signs of infection (evidenced by fever, trembling, and weak respiration) were recorded daily for a period of 2 weeks.

### 2.6. Lung Virus Titers

Lungs of the infected mice treated with various concentrations of BA were removed on days 3, 5, and 7. Viral titers in lungs were determined using a modified MDCK cell plaque assay [15]. Mice lungs were harvested into 1 mL DMEM media, homogenated, serially diluted, and added to duplicate confluent monolayers of MDCK cells for 1 h at 37°C. Each well was then covered with 1 mL of agar overlay (DMEM plus 0.2% BSA, 2 mg/mL NaHCO_3_, 2 mM Hepes, PSG, 0.5% agar, 0.01% DEAE dextran, and 0.5 *μ*g/mL trypsin; Sigma-Aldrich). After 2-3 days of incubation at 37°C, cells were fixed with 0.5 mL of Carnoy's fixative and stained with 2% crystal violet in 20% ethanol (Sigma-Aldrich). PFU/mL = (mean number of plaques/0.1) × (1/dilution factor) [14].

### 2.7. Histology

On day 7 after infection, mice were injected with 3 mg pentobarbitone and exsanguinated. The trachea was exposed and 1 mL of 2% formalin was instilled into the lungs. The inflated lungs were removed and placed in tubes containing 5 mL of 2% formal saline. Samples were embedded in paraffin and sections were stained with hematoxylin and eosin for histological analysis [16].

### 2.8. ELISA

IFN-*γ* in the mice serum and cell culture supernatants was detected by double layer ELISA. A 96-well plate was coated with antibody against IFN-*γ* (1 : 2,000; Santa Cruz, Goat) in 50 *μ*L PBS/well for 1 h at 37°C. The wells, after three successive washings with PBS, were incubated with 0.25% BSA and 0.05% Tween 20 in PBS for 30 min. Cell culture supernatants serially diluted (from 1 : 10) with PBS containing 0.25% BSA and 0.05% Tween 20 were added to the wells and incubated for 1 h at 37°C. After washing, 0.25% BSA and 0.05% Tween 20 in PBS were again added to each well and incubated for 10 min at 37°C. Anti-IFN-*γ*-HRP (1 : 5,000; Santa Cruz, Rabbit) was added and the plate incubated for 1 h at 37°C. After final washing, the plate was developed with *ο*-phenylenediamine in a buffer containing 0.012% hydrogen peroxide, 0.1 M citric acid, and 0.1 M Na_2_HPO_4_. The plates were then read at 570 nm and the sample concentrations were determined from a standard curve [17].

### 2.9. ELISPOT

We coated 96-well nitrocellulose-based plates (MAHA S45; Millipore) with 5 *μ*g of anti-mouse IFN-*γ* mAb/mL diluted in carbonate buffer (100 *μ*L/well). The plates were kept overnight at 4°C. Wells were then blocked with AIMV containing GSP for 1 h at 35°C. The cells were resuspended in AIMV and added to the wells (1 × 10^5^ cells/50 *μ*L per well, in triplicate for each condition) and incubated for 24 h at 37°C in 5% CO_2_. After incubation, the plates were extensively washed first with PBS and then with PBS-0.05% Tween (PBST) to detach and remove cells. Individual wells were then treated with 100 *μ*L of biotin-conjugated anti-mouse IFN-*γ* (1 *μ*g/mL), and the plates were incubated for 2 h at room temperature (RT). The plates were washed with PBST and then incubated with streptavidin-peroxidase (0.5 *μ*g/mL; Southern Biotechnology) for 1 h at RT. After an extensive washing with PBST and PBS, spots corresponding to IFN-*γ* producing cells were visualized with 3-amino-9-ethylcarbazole in 0.1 M sodium acetate (pH = 5.0). Spots were counted with an automated ELISPOT plate counter (Microvision Instruments), and values corresponded to the mean number of spots (in triplicate) for 1 × 10^6^ cells. Final values were obtained after subtraction of the control values (without stimulation). The mean value for negative controls according to the different experiments was a maximum of 1 spot/well, corresponding to 10 spots/million cells [18].

### 2.10. RT-PCR Analysis

Total RNA (2.5 *μ*g) was extracted using Trizol Reagent (Life Technologies, Inc.), and then RNA was reverse-transcribed with 20 U of reverse transcriptase using the Sensiscript RT Kit (Qiagen) with oligo (dT) primer [19]. After reverse transcription, 1 *μ*L of each reverse transcription reaction was used for PCR with Taq Polymerase (TaKaRa). The specific primers used to RT-PCR of A/PR/8/34, mice IFN-*γ*, mice GAPDH, human IFN-*γ*, and human GAPDH were as follows: 5′-GAT TGG TGG AAT TGG ACG AT-3′/5′-AGA GCA CCA TTC TCT CTA TT-3′ [20]; 5′-GAG AAA GAA GTC CTT GTG C-3′/5′-TCT ATC ATT CCA GTC CAT CCC-3′ [12]; 5′-GAC ATC AAG AAG GTG GTG AAG C-3′/5′-TGG AAA TTG TGA GGG AGA TGC-3′ [12]; 5′-TCC CAT GGG TTG TGT GTT TA-3′/5′-AAG CAC CAG GCA TGA AAT CT-3′ [21]; 5′- GAC ATC AAG AAG GTG GTG AA -3′/5′- TGT CAT ACC AGG AAA TGA GC -3′ [21], respectively. The PCR products, predicted as 247 bp of A/PR/8/34, 530 bp of mice IFN-*γ*, 336 bp of mice GAPDH, 198 bp of human IFN-*γ*, and 178 bp of human GAPDH in size, were separated on 1% agarose gel by electrophoresis.

### 2.11. Western Blotting Analysis

Whole cells and nuclear extracts were prepared using lysis buffer (1x PBS, 1% Nonidet P-40, 0.5% sodium deoxycholate, 150 mM NaCl, and 0.1% SDS) or lysate composed of the following: 20 mM HEPES (pH = 7.9); 420 mM NaCl; 1.5 mM MgCl_2_; 0.2 mM EDTA; 25% glycerol (v/v); 10 mM sodium molybdate; 1.0 mM DTT, 50 *μ*g/mL pepstatin, 25 *μ*g/mL aprotinin, 25 *μ*g/mL leupeptin, and 1.0 mM PMSF. Protease inhibitors and DTT were added to the lysate just before use. Proteins were separated on 12.5% SDS-PAGE under reducing conditions and transferred to polyvinylidene difluoride membrane. In indirect staining, mAbs against IFN-*γ*, JAK-1, JAK-2, phosphotyrosine 4G10 (Upstate Biotechnology), phosphor-JAK-2-Y1007/Y1008 (Santa Cruz Biotechnology), STAT-1, phosphor-STAT-1 (tyrosine 701), and phosphor-STAT-1 (serine 727) (Cell Signaling Technology) were used as primary antibodies, and goat anti mouse IgG-Dylight488 (Dako) was used as secondary antibody.

### 2.12. Experimental Infection and Treatment of PBMC

PBMC infected with A/PR/8/34 were collected by centrifugation, suspended in PBS, recentrifuged, and then suspended in 1 mL of infected allantoic fluid diluted to provide a multiplicity of 10 to 20 plaque-forming units per cell. The cultures were rotated gently for 60 min at RT during adsorption. After virus adsorption, the inoculum was removed from the cells, the cultures were washed once with PBS, and then PBMC were suspended in 1 mL of medium (1 × 10^6^ to 2 × 10^6^ cells per mL). A/PR/8/34 infected PBMC were treated with gradient of concentration of BA at 0 *μ*M, 0.1 *μ*M, 1 *μ*M, 10 *μ*M, 100 *μ*M, and 1 mM for 48 h at 37°C.

### 2.13. Oligodeoxynucleotide Treatment of Cells

The oligodeoxynucleotides were the same as described [22–25] and were as follows: oligo I, 5′-GGG GTT GGT TGT GTT GGG TGT TGT GT-RNH_2_; oligo II, 5′-AC ACA ACA CCC AAC ACA ACC AAC CCC-RNH_2_. PBMC infected with A/PR/8/34 were treated with (a) PBS, (b) 1 mM BA, (c) 1 mM BA and 25 *μ*M oligo I, and (d) 1 mM BA and 25 *μ*M oligo II. Viral titers were detected at 12 h, 24 h, 36 h, 48 h, 60 h, and 72 h.

### 2.14. Viral Titers Detection

Viruses in the PBMC culture supernatants were collected and then titrated by use of an MDCK plaque assay described previously [14, 15]. The percent inhibition rate was calculated by using the following formula: % Inhibition = (1 − Infectivity titer of BA treated virus/infective titer of nontreatment virus) × 100%.

### 2.15. Statistical Analysis

All experiments were performed at least three times and the results are from representative experiments. The statistical significance was determined using Student's *t*-test. Survival probability statistics were used to analyze the survival rates. Data were regarded as statistically significant at *P* < 0.01.

## 3. Results

### 3.1. Antiviral Activity of BA in Treating A/PR/8/34-Infected Mice

As described previously, we found that both the compound extract of* Scutellaria* (BA ≥ 7%) and pure BA exhibited an inhibitory activity on mouse-adapted A/FM/1/47 influenza virus A [12, 13]. To further systematic investigate the anti-influenza activity of BA, we examined its effect on mouse-adapted strain of human influenza virus A/PR/8/34 in mice.

**Figure 1:**
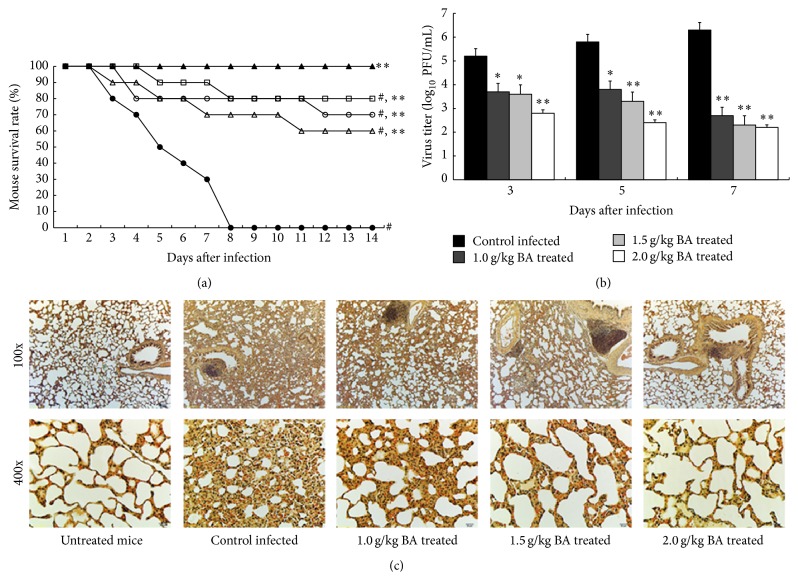
Antiviral activity of BA in treating A/PR/8/34-infected mice. Mice were inoculated with virus intranasally and treated with BA at the indicated concentration every 24 h for 14 days. Each group consists of 10 mice. Survival rate (a) of mice was observed in the next two weeks. ▲, untreated mice; ●, control A/PR/8/34-infected mice; ∆, 1.0 g/kg BA treated mice; ○, 1.5 g/kg BA treated mice; □, 2.0 g/kg BA treated mice. (b) Lungs of mice were homogenized separately, diluted, and centrifuged on the third, fifth, and seventh day after infection, and the EID_50_s were determined using 10 days' chick embryo. (c) Lungs of mice were removed and examined pathologically using HE staining on the seventh day after infection. ^#^Levels of significance of *P* < 0.05 against untreated mice; ^*∗*^ *P* < 0.05 against control infected mice; ^*∗∗*^ *P* < 0.01 against control infected mice.

We inoculated 4–6-week-old C57BL/6 mice with 0.1 LD_50_ (5 × 10^3^ PFU/mL) of A/PR/8/34 in 100 *μ*L PBS. All the A/PR/8/34 infected mice without BA treatment died on the 8th day following infection. After being treated with 1.0 g/kg, 1.5 g/kg, and 2.0 g/kg BA, 70, 80, and 80 percent of the infected mice survived to the 8th day following infection, respectively. Moreover, the survival rate in the BA treatment groups was found for 60, 70, and 80 percent 14 days after infection, which were significantly higher than those for mice without BA treatment (*P* < 0.01, Figure 1(a)). Meanwhile, A/PR/8/34-infected mice without BA treatment lost 15.66 ± 2.88% of their body weight, whereas those in the BA treatment group maintained or increased their weight.

In addition, in the untreated mice, virus titers increased to 10^6.3^ PFU/mL on the 7th day. In contrast, virus titers in the 1.0 g/kg, 1.5 g/kg, and 2.0 g/kg BA treated mice lungs decreased to 10^2.7^, 10^2.3^, and 10^2.2^ PFU/mL, respectively (Figure 1(b)). Furthermore, hemagglutination titers of the untreated mice lungs were 1 : 640, obviously higher than 1 : 80 in the BA treatment groups, indicating that BA has an inhibitory activity on A/PR/8/34 replication in mice lungs. We observed that the C57BL/6 wild type mice's lungs of the infected and untreated mice swelled and became red due to congestion. However, lungs of the infected mice treated with BA remained grayish white and were not congested. In the further study, performed by histological analysis, we observed that BA was able to resist A/PR/8/34 infection through regulating inflammatory responses, so as to prevent the formation of pulmonary fibrosis. Meanwhile, BA could also inhibit inflammatory lesion in mice lung tissues after A/PR/8/34 infection (Figure 1(c)).

### 3.2. Effects of BA on IFN-*γ* Production in A/PR/8/34-Infected Mice

In the previous study, we observed that the compound extract of* Scutellaria* (BA ≥ 7%) had an effect on inducing IFN-*γ* secretion in mice [12]; however, the effect of pure BA on IFN-*γ* production remains unclear. Thus, we further investigate the effects of BA on IFN-*γ* production in mice PBMC, and mice IFN-*γ* producing cells, including CD4^+^ and CD8^+^ T cells and NK cells.

**Figure 2:**
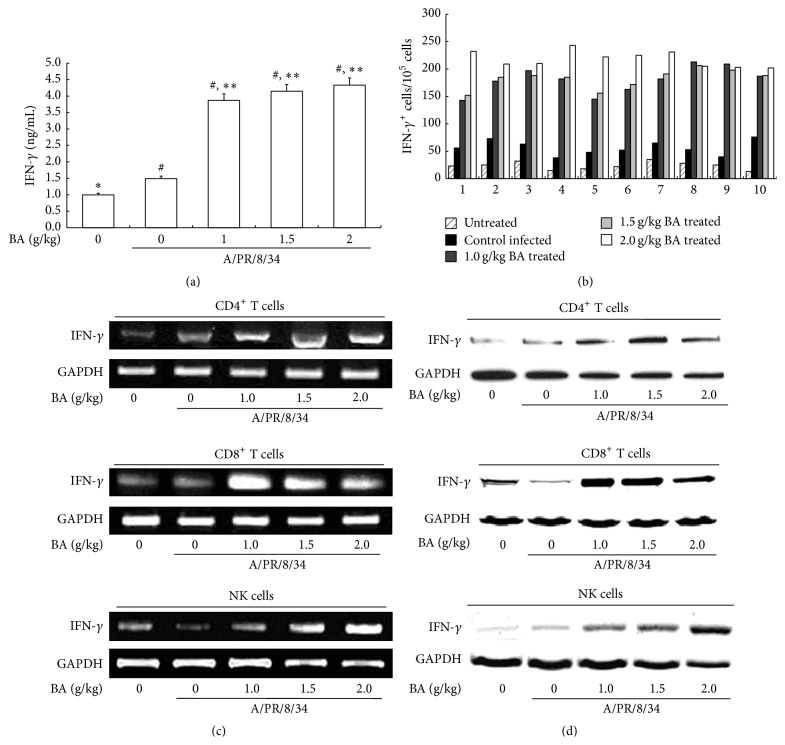
Effects of BA on IFN-*γ* production in A/PR/8/34-infected mice. Mice were treated as described in Figure 1. PBMC, CD4^+^, and CD8^+^ T cells and NK cells were then sorted on the seventh day. (a) 7 days after infection, IFN-*γ* in mice serum was calculated by ELISA, and the concentrations were determined from a standard curve. (b) Spot numbers of IFN-*γ* positive cells were obtained with PBMC from mice survived on the seventh day. 1 × 10^5^ cells were used per well, and experiments were performed in triplicate for each condition. (c) Total RNA was isolated from CD4^+^ and CD8^+^ T cells and NK cells and subjected to RT-PCR. The RT-PCR products of mice IFN-*γ* were analyzed on 1% agarose gel electrophoresis. (d) The cytosol protein was obtained from mice sorted CD4^+^ and CD8^+^ T cells and NK cells and analyzed by Western Blotting. ^#^Levels of significance of *P* < 0.05 against untreated mice; ^*∗*^ *P* < 0.05 against control A/PR/8/34-infected mice; ^*∗∗*^ *P* < 0.01 against control infected mice.

First, we detected the concentration of IFN-*γ* in the mice serum on day 7 by ELISA and found that BA could obviously induce IFN-*γ* secretion (*P* < 0.01, Figure 2(a)). Then, we evaluated the numbers of IFN-*γ* positive cells in mice PBMC using ELISPOT. As shown in Figure 2(b), IFN-*γ* positive cells significantly increased in the PBMC of mice treated with 1.0 g/kg, 1.5 g/kg, and 2.0 g/kg BA, compared to the control A/PR/8/34 infected mice and untreated mice. Thereafter, we focused our study on the mice IFN-*γ* producing cells, including CD4^+^ and CD8^+^ T cells and NK cells. RT-PCR results showed that the IFN-*γ* transcripts in the mice CD4^+^ and CD8^+^ T cells and NK cells increased in the BA treatment group (Figure 2(c)). Meanwhile, the production of IFN-*γ* also increased significantly in CD4^+^ and CD8^+^ T cells and NK cells in the BA treated mice (Figure 2(d)). Collectively, we found that BA could generally enhance IFN-*γ* synthesis and secretion in the IFN-*γ* producing cells in mice PBMC, including CD4^+^ and CD8^+^ T cells and NK cells.

### 3.3. Effects of BA in Treating A/PR/8/34-Infected IFN-*γ* or IFN-*γ* Receptor KO Mice

**Figure 3:**
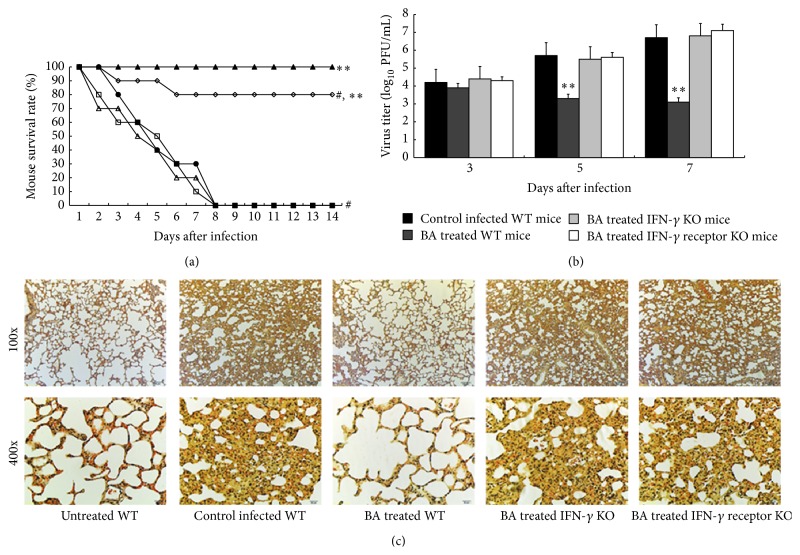
Effects of BA in treating A/PR/8/34-infected IFN-*γ* or IFN-*γ* receptor KO mice. Mice were inoculated with virus intranasally and treated with 2.0 g/kg BA every 24 h for 14 days. Each group consists of 10 mice. Survival rate (a) of mice was observed in the next two weeks. ▲, untreated WT mice; ●, control A/PR/8/34-infected WT mice; ○, BA treated WT mice; ∆, BA treated IFN-*γ* KO mice; □, BA treated IFN-*γ* receptor KO mice. (b) Lungs of mice were homogenized separately, diluted, and centrifuged on the third, fifth, and seventh day after infection, and the EID_50_s were determined using 10 days' chick embryo. (c) Lungs of mice were removed and examined pathologically using HE staining on the seventh day after infection. ^#^Levels of significance of *P* < 0.05 against untreated mice; ^*∗*^ *P* < 0.05 against control infected mice; ^*∗∗*^ *P* < 0.01 against control infected mice.

Due to our preliminary study, IFN-*γ* might be responsible for the anti-influenza virus activity of BA [13]. To confirm our hypothesis, we evaluated whether BA has a protective effect in IFN-*γ* (or receptor) knockout mice. We inoculated 4–6-week-old mice with 0.1 LD_50_ (5 × 10^3^ PFU/mL) of A/PR/8/34 in 100 *μ*L PBS. As we expected, all infected IFN-*γ*  −/− mice or IFN-*γ* receptor −/− mice treated with 2.0 g/kg BA died on the 8th day following infection (Figure 1(a)). In contrast, the survival rate in the 2.0 g/kg BA treated WT mice was found for 80 percent 14 days after infection (*P* < 0.01, Figure 3(a)). In addition, in the IFN-*γ* and IFN-*γ* receptor KO mice, virus titers increased to 10^6.8^ and 10^7.1^ PFU/mL, respectively, on the 7th day, even higher than that in the control infected WT mice. In contrast, virus titers in the 2.0 g/kg BA treated mice decreased to 10^3.1^ on day 7 after infection (Figure 3(b)). Furthermore, we observed that BA could not inhibit A/PR/8/34 infection in the IFN-*γ* (or receptor) KO mice, which suggests that the antiviral activity of BA is associated with IFN-*γ* (Figure 3(c)).

### 3.4. Antiviral Activity of BA on A/PR/8/34 Replication in Human PBMC through Induction of IFN-*γ*

To confirm the anti-A/PR/8/34 virus activity of BA in human, we obtained PBMC from healthy volunteers. Then, we infected human PBMC with A/PR/8/34 and treated the infected PBMC with gradient concentration of BA at 0 *μ*M, 0.1 *μ*M, 1 *μ*M, 10 *μ*M, 100 *μ*M, and 1 mM for 48 h at 37°C, to observe the inhibitory effect of BA on influenza virus.

**Figure 4:**
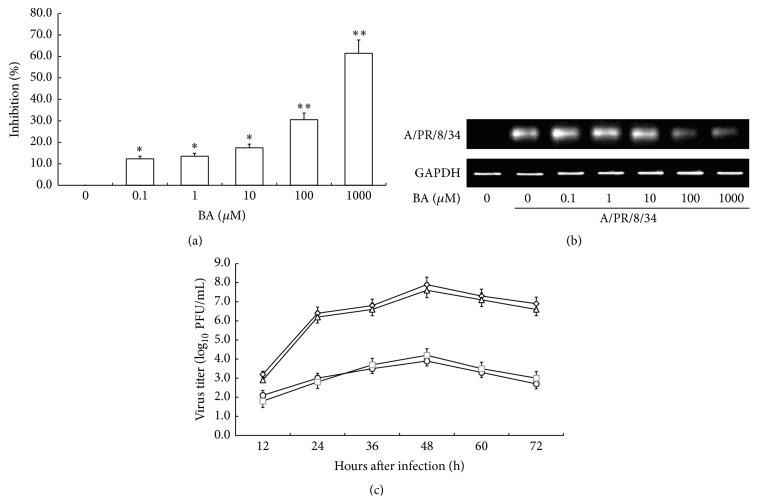
Antiviral activity of BA on A/PR/8/34 replication in human PBMC. A/PR/8/34 infected PBMC were treated with gradient concentration of BA at 0 *μ*M, 0.1 *μ*M, 1 *μ*M, 10 *μ*M, 100 *μ*M, and 1 mM for 48 h at 37°C. (a) Viruses in the culture supernatants were collected and then titrated by use of an MDCK plaque assay to determine the virus titers. The percent inhibition rate was calculated by using the following formula: % Inhibition = (1 − Infective titer of BA treated virus/infective titer of nontreatment virus) × 100%. (b) Total RNA was isolated from PBMC and subjected to RT-PCR. The RT-PCR of A/PR/8/34 was analyzed on 1% agarose gel electrophoresis. (c) Viral titers of A/PR/8/34 in PBMC treated with PBS (⋄), 1 mM BA (○), 1 mM BA with 25 *μ*M oligo I (□), and 1 mM BA with 25 *μ*M oligo II (△) were determined at 12, 24, 36, 48, 60, and 72 h.p.i. Each data shows mean ± SE of three respective determinations.  ^*∗*^Levels of significance of *P* < 0.05 against control infected PBMC,  ^*∗∗*^levels of significance of *P* < 0.01 against control infected PBMC cells.

To evaluate the anti-A/PR/8/34 virus activity of BA* in vitro*, we determined the virus titers by use of an MDCK plaque assay. As shown in Figure 4(a), a dose-dependent inhibition of A/PR/8/34 virus was observed after treating the infected PBMC with BA. Presence of 100 *μ*M BA in PBMC yielded 30% inhibition and about 60% was inhibited by 1 mM BA (*P* < 0.01). In addition, the replication of A/PR/8/34 was significantly inhibited in the presence of 100 *μ*M and 1 mM BA (Figure 4(b)). To further assess the antiviral activity of BA* in vitro*, we examined the virus growth kinetics in PBMC treated with or without 1 mM BA. A/PR/8/34 in PBMC treated with 1 mM BA grew significantly worse than nontreatment. The peak viral titer reached 10^3.9^ PFU/mL at 48 hours after infection (h.p.i.) for A/PR/8/34 treated with 1 mM BA, compared to 10^7.9^ PFU/mL at 48 h.p.i. for nontreatment (*P* < 0.01, Figure 4(c)). In addition, using the high affinity IFN-*γ* aptamers, we found that the antiviral activity of 1 mM BA was significantly suppressed by 25 *μ*M oligo I, taking oligo II as negative control (Figure 4(c)).

**Figure 5:**
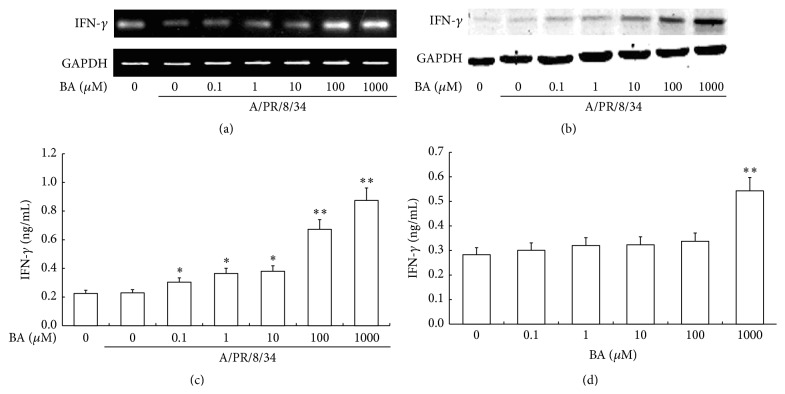
Effects of BA on IFN-*γ* production in human PBMC. A/PR/8/34 infected PBMC were treated with gradient concentration of BA at 0 *μ*M, 0.1 *μ*M, 1 *μ*M, 10 *μ*M, 100 *μ*M, and 1 mM for 48 h at 37°C. (a) Total RNA was isolated from PBMC and subjected to RT-PCR. The RT-PCR of human IFN-*γ* products was analyzed on 1% agarose gel electrophoresis. (b) The cytosol proteins were obtained from PBMC and analyzed by Western Blotting with anti-IFN-*γ* antibody. (c) IFN-*γ* production in cell culture supernatant infected with (c) or without (d) A/PR/8/34 virus infection was calculated by ELISA, and the results were determined from a standard curve.  ^*∗*^Levels of significance of *P* < 0.05,  ^*∗∗*^levels of significance of *P* < 0.01.

Meanwhile, we tested the IFN-*γ* synthesis in human PBMC after different treatment and found that BA was able to enhance IFN-*γ* transcription at a high concentration of 100 *μ*M to 1 mM under A/PR/8/34 virus infection (Figure 5(a)). In the further examination, we observed the effect of BA on IFN-*γ* production in the cytosol protein of PBMC by Western Blotting. As shown in Figure 5(b), there was no significant difference in IFN-*γ* production in PBMC after being treated with 0.1 *μ*M to 10 *μ*M BA. However, BA, at a high concentration of 100 *μ*M to 1 mM, had an obvious influence on IFN-*γ* protein expression, similar to the transcriptional level. Then, we investigated the secretion of IFN-*γ* in PBMC cell culture supernatants. As a result, we found that the influence of BA on IFN-*γ* secretion exhibited a dose-dependent manner; BA was able to increase IFN-*γ* secretion significantly at a concentration from 100 *μ*M to 1 mM under A/PR/8/34 virus infection (Figure 5(c)). Interestingly, when human PBMC were treated with BA without A/PR/8/34 virus infection, only 1 mM BA can significantly enhance the IFN-*γ* secretion in the cell culture supernatants (Figure 5(d)).

**Figure 6:**
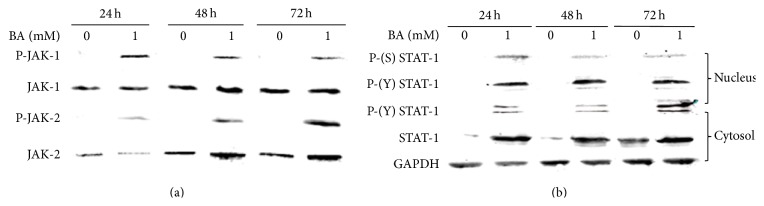
Effects of BA in JAK/STAT-1 pathway. Human PBMC were treated with or without 1 mM BA for 24 h, 48 h, and 72 h at 37°C. (a) The cytosol protein was separated by electrophoresis in a 12.5% SDS-polyacrylamide gel and transferred to a nitrocellulose membrane. The proteins were blotted with anti-JAK-1, anti-JAK-2, anti-phosphotyrosine 4G10, and anti-phospho-JAK-2-Y1007/Y1008 Abs, respectively. (b) The nucleus proteins (upper panels) and cytosol proteins (lower panels) were obtained and analyzed by Western Blotting using Abs against STAT-1, phosphor-STAT-1 (tyrosine 701), and phosphor-STAT-1 (serine 727).

IFN-*γ* possesses antiviral and immunomodulatory activities and mediates intracellular effects via the JAK/STAT-1 pathway. Binding of IFN-*γ* to its receptor results in activation of JAK-1 and JAK-2 through the phosphorylation of tyrosine 1022/1023 on JAK-1 and tyrosine 1007/1008 on JAK-2, respectively [26, 27]. To determine whether this step in the JAK/STAT-1 pathway is activated, the expression and phosphorylation of the JAKs in human PBMC were assessed. As shown in Figure 6(a), Tyrosine phosphorylation of JAK-1 and JAK-2 was detected within 24 h and sustained for up to 72 h in human PBMC after being treated with 1 mM BA. Transcriptional activation of genes regulated by the JAK-STAT-1 pathway requires phosphorylation of tyrosine residue 701 on STAT-1 by the JAKs [28, 29]. The tyrosine phosphorylation of JAK-1 and JAK-2 prompted us to examine the expression and phosphorylation status of STAT-1. Over the 72 h time course, total STAT-1 levels increased in human PBMC treated with 1 mM BA. More importantly, higher levels of P-Y^701^-STAT-1 were detected at 24 h, 48 h, and 72 h, compared to nontreatment control. Moreover, in the dynamic analysis of STAT-1 phosphorylation after BA treatment, STAT-1 phosphorylation at tyrosine 701 and serine 727 was enhanced in nucleus as well (Figure 6(b)). Collectively, we indicated that BA activated JAK/STAT-1 signaling pathway in human PBMC as an inducer of IFN-*γ*. These results indicated that BA could inhibit A/PR/8/34 replication in human PBMC through IFN-*γ*.

### 3.5. Effects of BA on IFN-*γ* Production in Human CD4^+^ and CD8^+^ T Cells and NK Cells

In the above study, we found that BA could inhibit A/PR/8/34 replication* in vitro* and enhance IFN-*γ* production in human PBMC significantly at a concentration of 1 mM. To investigate the target cells of BA, we examined the effects of 1 mM BA on IFN-*γ* synthesis and secretion in human IFN-*γ* producing cells, including CD4^+^ and CD8^+^ T cells and NK cells.

Cell viability was examined to investigate the potential activity of BA on human IFN-*γ* producing cells. We found that BA had no significant influence on cell viability in CD4^+^ and CD8^+^ T cells and NK cells, even at the concentration of 1 mM. Therefore, in the following experiment, the effects of BA on IFN-*γ* production were not the result of possible cell viability effects on these cells.

**Figure 7:**
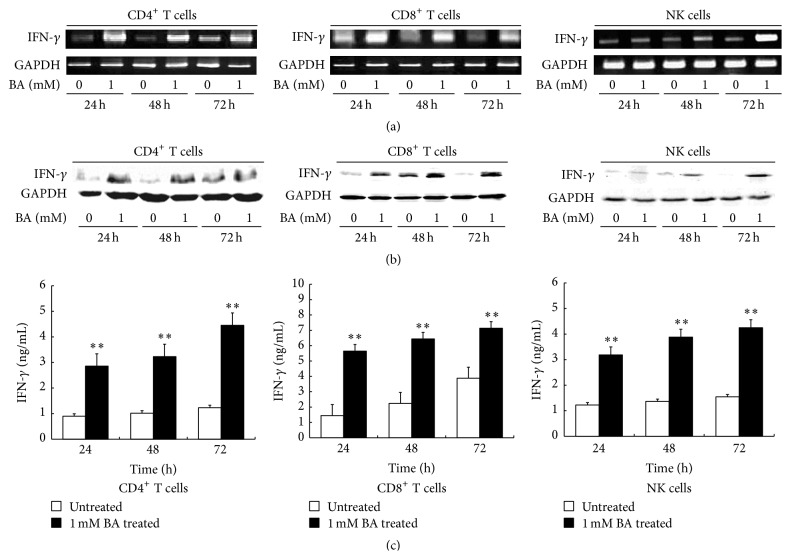
Effects of BA on IFN-*γ* production in human CD4^+^ and CD8^+^ T cells and NK cells. Human PBMC sorted CD4^+^ and CD8^+^ T cells and NK cells were treated with or without 1 mM BA for 24 h, 48 h, and 72 h. (a) Total RNA was isolated from CD4^+^ and CD8^+^ T cells and NK cells and subjected to RT-PCR. The RT-PCR products of human IFN-*γ* were analyzed on 1% agarose gel electrophoresis. (b) The cytosol protein was obtained and analyzed by Western Blotting using anti-human IFN-*γ* antibody. (c) Cell culture supernatants were obtained and analyzed by ELISA; results were expressed as the sample concentrations, determined from a standard curve. Each data shows mean ± SE of three respective determinations.  ^*∗∗*^Levels of significance of *P* < 0.01 against untreated cells.

In order to investigate the effects of BA on IFN-*γ* transcription in CD4^+^ and CD8^+^ T cells and NK cells, we harvest cells treated or untreated with 1 mM BA for 24 h, 48 h, and 72 h. Total RNA was isolated and subjected to RT-PCR with specific human IFN-*γ* primers. The results shown in Figure 7(a) represented that BA promote IFN-*γ* mRNA expression in CD4^+^ and CD8^+^ T cells and NK cells and exhibited a continuous effect for 24 h to 72 h. We next examined whether BA could enhance IFN-*γ* protein expression in the cytosol protein. Western Blotting results showed that BA promoted IFN-*γ* protein synthesis in CD4^+^ and CD8^+^ T cells and NK cells in the cytosol protein and exhibited a sustained action for 24 h to 72 h, similar to the transcriptional level (Figure 7(b)). In the study of IFN-*γ* secretion, we used ELISA assay to evaluate its concentration in the culture supernatants of CD4^+^ and CD8^+^ T cells and NK cells treated with 1 mM BA. Untreated cell culture supernatants were used as negative control. The results showed that the IFN-*γ* protein in the supernatants increased for 24 h to 72 h, and the treated group was kept at a higher level, compared to untreated group (*P* < 0.01, Figure 7(c)), which indicated that BA was able to induce IFN-*γ* secretion. In summary, we proved that BA was able to enhance IFN-*γ* synthesis and secretion in human CD4^+^ and CD8^+^ T cells and NK cells.

## 4. Discussion

Influenza is still a major health burden, and options for the control and treatment of the disease are limited, because of the high degree of virus variability, requiring annual vaccination [30]. Thus, there is an urgent public health need to develop effective drugs against various influenza viruses.

In eastern countries,* Scutellaria baicalensis* Georgi has been used to treat influenza for centuries and proved to be effective and safe, but the antiviral mechanism remains unknown. Thus, we first extracted the compound of* Scutellaria baicalensis* Georgi and examined its antiviral activity against a mouse-adapted influenza virus A/FM/1/47. As we expected, the compound had an effect on inhibiting influenza virus replication in mice; meanwhile, we found that this compound might also induce IFN-*γ* secretion [12]. It is known that the compound isolated from* Scutellaria baicalensis* Georgi contains four major flavones: Wogonin, Wogonoside, Baicalein, and BA with ratios to the dry material about 1.3%, 3.55%, 5.41%, and 10.11%, respectively [31]. As a main component in* Scutellaria baicalensis* Georgi, BA has a variety of beneficial effects including antiviral activities [8]. In particular, the anti HTLV-1 and HIV-1 effect of BA have been documented [9–11]. However, the role of BA in anti-influenza virus activity has not been extensively studied. In the further study, we identified the inhibitory effect of BA on A/FM/1/47 replication in mice for the first time and demonstrated that BA could significantly induce IFN-*γ* secretion in mice serum [12, 13]. Thus, we believe that IFN-*γ* plays an important role in the antiviral action of BA.

Based on the previous research, we first identified the antiviral activity of BA in treating A/PR/8/34-infected mice and observed that BA was able to attenuate A/PR/8/34 associated death and inhibit virus replication in mice lungs. To verify that IFN-*γ* is responsible for the antiviral activity of BA, we tested whether BA could protect IFN-*γ*  −/− mice and IFN-*γ* receptor −/− mice from A/PR/8/34 infection. As expected, BA possessed no anti-influenza virus effect in IFN-*γ* (or receptor) knockout mice, which prompted that the antiviral activity of BA is associated with its effect on IFN-*γ* production.

The IFN-*γ* is a pleiotropic cytokine mainly secreted by CD4^+^ and CD8^+^ T cells and NK cells and has been believed to be a first line of host defense in the control of viral infections [32]. Considering the antiviral activity of BA correlated with IFN-*γ*, we focused our study on its IFN-*γ* inducing action. In evaluating the IFN-*γ* secretion in mice serum, significant increases of IFN-*γ* were detected in the mice treated with BA at an effective concentration. In addition, BA could also increase the percent of IFN-*γ* positive cells in mice PBMC. Since CD4^+^ and CD8^+^ T cells and NK cells are the major IFN-*γ* producing cells in PBMC, we wondered if BA could directly induce IFN-*γ* production in all these cells. As expected, the transcription of IFN-*γ* mRNA increased in mice CD4^+^ and CD8^+^ T cells and NK cells, which results in the increases of IFN-*γ* production. These findings prompted a general action of BA on inducing IFN-*γ* synthesis in mice IFN-*γ* producing cells.

To further validate these effects of BA in human PBMC, we first used 0.1 *μ*M to 1 mM BA to investigate its inhibitory actions on A/PR/8/34 infection* in vitro*. Similarly to the results* in vivo*, BA exhibited anti-A/PR/8/34 replication activity in a dose-dependent manner and significantly reduced A/PR/8/34 infective titers at a concentration of 1 mM. More importantly, the antiviral activity of BA was suppressed by the high affinity IFN-*γ* aptamers, which confirmed that IFN-*γ* is responsible for the antiviral activity of BA [33]. Meanwhile, BA could apparently enhance IFN-*γ* production in human PBMC, especially at 100 *μ*M to 1 mM. Since JAK/STAT-1 signaling pathway plays an important role in IFN-*γ* activation, we then examined the representative changes during the activation process. We found that the JAK/STAT-1 pathway in human PBMC was activated in the treatment of 1 mM BA.

According to the general IFN-*γ* inducing action of BA in mice, we further confirmed the effects of BA in human CD4^+^ and CD8^+^ T cells and NK cells using 1 mM BA. Several methods were used to verify the activity of BA on inducing IFN-*γ* expression and secretion. As a result, BA could enhance IFN-*γ* production at 1 mM in human CD4^+^ and CD8^+^ T cells and NK cells and possessed a sustained action for 24 h to 72 h. Recent studies indicated that the action of BA in inducing IFN-*γ* production of T cells might be mediated by TCR (*αβ*) [33]. Collectively, BA could generally induce IFN-*γ* production in mice and human IFN-*γ* producing cells, so as to resist various viruses, including influenza virus.

Two main problems of present therapy that play a major role in treatment of influenza are the high degree of virus variability and the reorganization of various viruses. Effective vaccine is important in the prevention of influenza; however, it could not be effective against new generated influenza virus. Thus, it is urgent to develop broad-spectrum antiviral drugs. In this report, we found that BA exhibited anti-influenza virus A (H_1_N_1_) activities* in vivo* and* in vitro* through upregulation of IFN-*γ* production in CD4^+^ and CD8^+^ T cells and NK cells. It has been determined that IFN is a strong activator, produced both in the early stages and later stages of viral infection, and plays an important role in innate immune response. Therefore, the antiviral activity of BA might not be restricted in the species of virus, which makes it a potent therapy in treating various influenza viruses. Moreover, BA, purified from* Scutellaria baicalensis* Georgi, has been used in the treatment of several diseases and proved to be safety [34]. Thus, development in the study of BA in antiviral activities may constitute a new safe approach for viral prevention and therapy.
