# Corrigendum to “Role of Baicalin in Anti-Influenza Virus A as a Potent Inducer of IFN-Gamma”

**DOI:** 10.1155/2022/9873924

**Published:** 2022-04-28

**Authors:** Ming Chu, Lan Xu, Ming-bo Zhang, Zheng-yun Chu, Yue-dan Wang

**Affiliations:** ^1^Department of Immunology, School of Basic Medical Sciences, Peking University, Beijing 100191, China; ^2^Key Laboratory of Medical Immunology, Ministry of Health, Beijing 100191, China; ^3^Pharmacy Departments, Liaoning University of Traditional Chinese Medicine, Liaoning 116600, China

In the article titled “Role of Baicalin in Anti-Influenza Virus A as a Potent Inducer of IFN-Gamma” [1], there appears to be an image duplication in Figure 1, as raised on PubPeer [2]. In Figure 1(c), the panel showing the 100x + untreated mice inadvertently duplicated the panel showing the 100x + 2.0 g/kg BA-treated mice.

The corrected figure, as approved by the editorial board, is shown below.

## Figures and Tables

**Figure 1:**
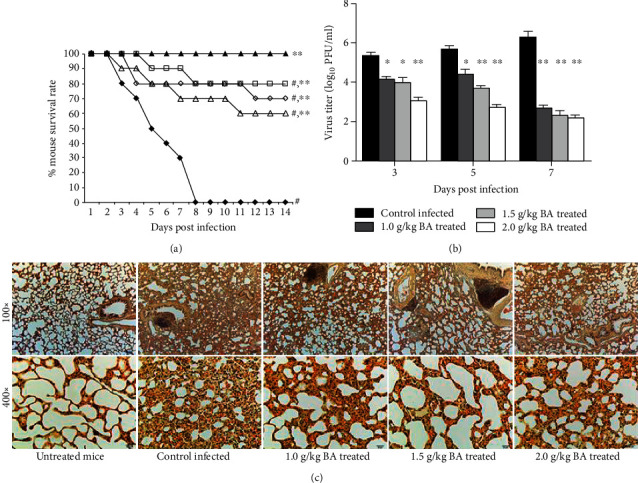
Antiviral activity of BA in treating A/PR/8/34-infected mice. Mice were inoculated with virus intranasally and treated with BA at the indicated concentration every 24 h for 14 days. Each group consists of 10 mice. (a) Survival rate of mice was observed in the next two weeks. ▲: untreated mice; ●: control A/PR/8/34-infected mice; ∆: 1.0 g/kg BA-treated mice; ○: 1.5 g/kg BA-treated mice; □: 2.0 g/kg BA-treated mice. (b) Lungs of mice were homogenized separately, diluted, and centrifuged on the third, fifth, and seventh day after infection, and the EID_50_s were determined using 10-day chick embryo. (c) Lungs of mice were removed and examined pathologically using HE staining on the seventh day after infection. ^#^Levels of significance of *P* < 0.05 against untreated mice; ^∗^*P* < 0.05  against control infected mice; ^∗∗^*P* < 0.01 against control infected mice.
